# Dynamics of Peripheral Blood Lymphocyte Subpopulations in the Acute and Subacute Phase of Legionnaires’ Disease

**DOI:** 10.1371/journal.pone.0062265

**Published:** 2013-04-30

**Authors:** Cornelis P. C. de Jager, Eugenie F. A. Gemen, Jacqueline Leuvenink, Mirrian Hilbink, Robert J. F. Laheij, Tom van der Poll, Peter C. Wever

**Affiliations:** 1 Department of Emergency Medicine and Intensive Care, Jeroen Bosch Hospital, ’s-Hertogenbosch, The Netherlands; 2 Department of Clinical Chemistry and Hematology, Jeroen Bosch Hospital, ’s-Hertogenbosch, The Netherlands; 3 Jeroen Bosch Academy, Jeroen Bosch Hospital, ’s-Hertogenbosch, The Netherlands; 4 Center of Infection and Immunity Amsterdam and Center of Experimental and Molecular Medicine, Academic Medical Center, University of Amsterdam, The Netherlands; 5 Department of Medical Microbiology and Infection Control, Jeroen Bosch Hospital, ’s-Hertogenbosch, The Netherlands; Blood Systems Research Institute, United States of America

## Abstract

**Study Objective:**

Absolute lymphocytopenia is recognised as an important hallmark of the immune response to severe infection and observed in patients with Legionnaires’ disease. To explore the immune response, we studied the dynamics of peripheral blood lymphocyte subpopulations in the acute and subacute phase of LD.

**Methods and Results:**

EDTA-anticoagulated blood was obtained from eight patients on the day the diagnosis was made through detection of *L. pneumophila* serogroup 1 antigen in urine. A second blood sample was obtained in the subacute phase. Multiparametric flow cytometry was used to calculate lymphocyte counts and values for B-cells, T-cells, NK cells, CD4^+^ and CD8^+^ T-cells. Expression of activation markers was analysed. The values obtained in the subacute phase were compared with an age and gender matched control group. Absolute lymphocyte count (×10^9^/l, median and range) significantly increased from 0.8 (0.4–1.6) in the acute phase to 1.4 (0.8–3.4) in the subacute phase. B-cell count showed no significant change, while T-cell count (×10^6^/l, median and range) significantly increased in the subacute phase (495 (182–1024) versus 979 (507–2708), p = 0.012) as a result of significant increases in both CD4^+^ and CD8^+^ T-cell counts (374 (146–629) versus 763 (400–1507), p = 0.012 and 119 (29–328) versus 224 (107–862), p = 0.012). In the subacute phase of LD, significant increases were observed in absolute counts of activated CD4^+^ T-cells, naïve CD4^+^ T-cells and memory CD4^+^ T-cells. In the CD8^+^ T-cell compartment, activated CD8^+^ T-cells, naïve CD8^+^ T-cell and memory CD8^+^ T-cells were significantly increased (p<0.05).

**Conclusion:**

The acute phase of LD is characterized by absolute lymphocytopenia, which recovers in the subacute phase with an increase in absolute T-cells and re-emergence of activated CD4^+^ and CD8^+^ T cells. These observations are in line with the suggested role for T-cell activation in the immune response to LD.

## Introduction

Legionnaires’ disease (LD) is a potentially fatal pneumonia mainly caused by *Legionella pneumophila* serogroup 1. These Gram-negative bacteria cause an intracellular infection of alveolar macrophages and are an important cause of community- and hospital-acquired pneumonia. Infection with *Legionella* species ranks among the three most common causes of severe community-acquired pneumonia (CAP) [1]. In the Netherlands, around 300 patients are admitted annually to the hospital with CAP due to *L. pneumophila* [2]. If not asymptomatic, the clinical presentation of LD may be variable, from relatively mild disease to severe pneumonia requiring Intensive Care Unit (ICU) admission with high mortality rates depending, among others, on the presence of multi-organ involvement and effectiveness of initial therapy [3], [4], [5]. Currently, immunochromatographic assays for rapid qualitative detection of *L. pneumophila* serogroup 1 antigen in urine specimens and polymerase chain reaction tests for detection of *L. pneumophila* DNA in serum and respiratory specimens allow for rapid diagnosis of LD [6], [7]. Yet, despite these advances in recognition of *L. pneumophila* infection, its clinical course remains unpredictable [8].

Absolute lymphocytopenia (peripheral blood lymphocyte count below 1.0×10^9^/l) is increasingly recognised as an important hallmark of the immune response to various microorganisms [9], [10]. This phenomenon has been observed in several forms of CAP, especially in the acute phase, and is probably limited to T-cells and T-cell subpopulations [11]. The severity of pneumonia may be correlated with the degree of lymphocytopenia in the acute stage as is observed in patients with *Mycoplasma pneumoniae* pneumonia [12], [13].

It has been hypothesized that depression of absolute peripheral blood T-cell counts in CAP patients represents shift of these cells towards the lung in order to be sequestered in protective mechanisms [14], [15]. In sepsis, proposed mechanisms of lymphocytopenia are margination and redistribution of lymphocytes within the lymphatic system and marked accelerated apoptosis [16], [17]. In apoptosis, selected cell populations are actively deleted from certain tissues and in animal sepsis models this has been shown a mechanism of lymphocyte death [18], [19], [20]. In septic patients, extensive apoptosis was noted in circulating lymphocytes occurring by both death receptor- and mitochondrial-mediated pathways [21].

Among laboratory findings on hospital admission in patients with LD, absolute lymphocytopenia was observed in the majority of patients [22], [23]. Furthermore, LD is characterized by accumulation of activated T-cells in the lungs [24]. This could well be a reflection of early recruitment of activated T-cells at the site of the infection. Increased serum levels of the Th1 cytokines interferon-γ and interleukin-12 suggest a predominance of the cellular immune response in patients with LD in the resolution of the primary infection [25]. Furthermore, animal models show that control of the infection and clearance of *L. pneumophila* depend on recruitment and function of CD4^+^ and CD8^+^ T-cells [26].

Cell-mediated immunity appears to be the primairy host defence mechanism against *Legionella pneumophila* infection, although exact immunopathogenesis in LD remains unknown.

To further explore the cellular immune response in patients with LD, we studied the proportions and dynamics of peripheral blood lymphocyte subpopulations in the acute phase, characterized by lymphocytopenia, and subacute phase of LD.

## Materials and Methods

### Patients

Adult patients (age 18 years and older) admitted over a 15-month period (2006–2007) with CAP caused by *L. pneumophila* serogroup 1 were studied. Community-acquired pneumonia (CAP) was defined as the presence of symptoms of lower respiratory tract infection (new cough, sputum production, dyspnoea, hypo- or hyperthermia, altered breath sounds upon physical examination) in the presence of a new infiltrate on plain chest radiography. Sputum culture, aerobic and anaerobic blood cultures, serologic analysis and immunochromatographic urinary antigen detection tests were routinely performed according to standard microbiological methods to identify potential pathogenic microorganisms such as among others *Streptococcus pneumoniae, Haemophilus influenzae, Moraxella catarrhalis, Staphylococcus aureus, Pseudomonas aeruginosa, Enterobacteriaceae, Mycoplasma pneumonia, Chlamydia psittaci* and *Legionella pneumophila*.

Patients were admitted to the Jeroen Bosch Hospital, an 800-bed teaching hospital in ’s-Hertogenbosch, the Netherlands. Ethylenediaminetetraacetic acid (EDTA)-anticoagulated blood from eight patients with LD was obtained on the day the diagnosis was made (acute phase) through detection of *L. pneumophila* serogroup 1 antigen in urine using an immunochromatographic assay (BinaxNOW *Legionella*, Binax, Inc., Scarborough, ME, USA). The case definition proposed by the European Working Group for *Legionella* Infections for confirmed cases was used in this study [27]. The validated CURB-65 score was calculated in all patients upon admission. The purpose of the CURB-65 score is to calculate the probability of mortality in patients with CAP [28]. Subsequent antibiotic treatment of patients was consistent with national guidelines and consisted of second (ciprofloxacin) or fourth generation (moxifloxacin) fluoroquinolones [29]. A second blood sample was obtained one week later or on the day of discharge from the hospital (subacute phase). Eight age and sex matched healthy volunteers who had no existing comorbidities were included as a control group.

### Ethics Statement

Individual patient consent was not obtained since blood samples used in this study were all drawn for routine hematological analysis by order of the treating physician. The Internal Review Board of the Jeroen Bosch Hospital approves anonymous use of discarded blood for scientific purposes. All patients who donate blood are informed of this possibility with right of refusal. The Internal Review Board of the Jeroen Bosch Hospital specifically approved the study and waived the need for informed consent.

### Infection Markers

Data on infection markers were retrieved from the hospital’s laboratory information system. C-reactive protein (CRP) levels were measured with a fully automated enzyme-linked immunoassay using an Aeroset 2.0 analyzer (Abbott Diagnostics, Santa Clara, CA, USA). White blood cell (WBC) counts and leucocyte differentiation were determined on a Sysmex XE-2100 hematology analyser (Sysmex Corporation, Kobe, Japan).

### Flow Cytometric Immunophenotyping

Four-color flow cytometric immunophenotyping was performed on the day of blood sampling using the lyse and wash whole-blood method. Aliquots of 50 µl EDTA blood were incubated for 15 minutes at room temperature in the dark with different combinations of the following monoclonal antibodies: CD3 conjugated to fluorescein isothiocyanate (FITC), CD27 FITC, HLA-DR FITC, CD2 conjugated to phycoerythrin (PE), CD16/56 PE, CD38 PE, CD45RA PE, HLA-DR PE, CD4 conjugated to peridinin chlorophyll protein-cyanine 5.5 (PerCP-Cy5.5), CD8 PerCP-Cy5.5, CD19 PerCP-Cy5.5, CD3 conjugated to allophycocyanin (APC) and CD4 APC. All antibodies were obtained from Becton Dickinson (BD), San Jose, CA, USA. After staining, erythrocytes were lysed with FACS Lysing Solution (BD) according to the manufacturer’s protocol. The remaining leucocytes were washed twice with 0.5% bovine serum albumin/phosphate-buffered saline. Analysis was performed using CellQuest ProSoftware (BD) on a FACSCalibur flow cytometer (BD), which was calibrated according to the guidelines of Kraan et al [30]. In each analysis, 20,000 events were acquired. The lymphocyte gate was defined by specific forward and side scatter properties. We calculated the absolute number of cells in a specific lymphocyte subpopulation by multiplying the absolute lymphocyte count expressed in ×10^6^/l units and the relative size of that lymphocyte subpopulation within the lymphocyte gate. Expression of activation and maturation markers was analysed on CD4^+^ and CD8^+^ T-cells. In the CD4^+^ T-cell compartment, activated (CD3^+^CD4^+^CD38^+^HLA-DR^+^), naïve (CD3^+^CD4^+^CD45RA^+^) and memory (CD3^+^CD4^+^CD45RA^−^) T-cells were analysed. In the CD8^+^ T-cell compartment, activated (CD3^+^CD8^+^CD38^+^HLA-DR^+^), naïve (CD3^+^CD8^+^CD45RA^+^CD27^+^), memory (CD3^+^CD8^+^CD45RA^−^) and effector (CD3^+^CD8^+^CD45RA^+^CD27^−^) T-cells were analysed.

### Statistical Analysis

We first judged for fit to the normal distribution by using stem-and-leaf plots and quantile-quantile plots. As our data did not follow a normal distribution, Wilcoxon signed rank tests were performed for the comparison of CRP levels, WBC counts, absolute neutrophil counts, absolute lymphocyte and lymphocyte subpopulations counts in the acute and subacute phase of LD. To test the differences in CRP levels, WBC counts, absolute neutrophil counts, absolute lymphocyte and lymphocyte subpopulations counts in the subacute phase of LD and healthy controls, Mann Whitney U tests were performed. Finally, we performed Wilcoxon signed rank tests to assess the differences between the relative increase of the absolute lymphocyte count and the relative decrease/increase of the various lymphocyte subpopulations (relative expansion). A p-value of less than 0.05 was considered statistically significant. All reported p-values are two sided. Statistical analyses were performed using SPSS software (SPSS, version 19, IBM, Chicago, Il, USA).

## Results

### Patients

During the study period ten patients with CAP due to *L. pneumophila* serogroup 1 were hospitalized. Two patients were not included in the analysis because subacute phase blood samples were not obtained. Thus, the study population consisted of eight patients with a median age of 56 years (range 31–93). None of the patients was known for a condition that could otherwise explain lymphocytopenia upon presentation (e.g. haematological disease, chemotherapy, malnutrition). None of the patients used glucocorticoid medication upon admission or received glucocorticoid treatment. In none of the patients a co-infection with another pathogen was detected. None of the patients were immunocompromised. The median hospital stay for all patients was seven days (range 5–14) days. Timely adequate antibiotic treatment (within 12 hours after admission) was achieved in 7/8 (87.5%) patients [31]. All patients survived their disease episode. The base-line characteristics of the study patients are shown in table 1. Eight age and sex matched healthy volunteers who had no existing comorbidities were included as a control group (median age 55, range 31–93).

**Table 1:** Baseline characteristics upon hospitalization of Legionnaires’ disease patients (n = 8).

Patient	Age	Gender	Temp	CRP	WBC-Count	Lymphocytecount	X-ray	AB within 12 hours	Riskfactors	Duration Symptoms	CURB-65	ICU	LOS	Survival
**1**	55	M	38.7	342	8.2	0.7	Infiltratecompleteright lung	Yes	Smoking	5 days	0	No	8	Yes
**2**	31	M	40.0	437	21.4	1.6	Infiltrate leftupper lobe	Yes	Smoking	4 days	0	No	7	Yes
**3**	49	F	39.4	284	17.5	1.0	BilateralInfiltrates	Yes	Smoking	4 days	1	No	14	Yes
**4**	46	M	39.2	256	11.3	1.0	Infiltrate rightlower lobe	Yes	Smoking	6 days	0	Yes	5	Yes
**5**	69	M	39.9	455	9.2	0.4	Infiltrate rightlower lobe	Yes	None	6 days	0	No	14	Yes
**6**	56	M	39.4	347	14.0	1.0	Infiltratecompleteright lung	Yes	None	4 days	0	No	6	Yes
**7**	93	F	37.9	420	12.7	0.4	Infiltrate rightlung	Yes	None	4 days	4	No	14	Yes
**8**	76	M	39.3	330	8.7	0.4	Infiltrate rightupper lobe	No	None	5 days	2	No	6	Yes
**Median (range)**	56 (31–93)	NA	39.3 (37.9–40.0)	345 (256–455)	12.0 (8.2–21.4)	0.8 (0.4–1.6)	NA	NA	NA	4 (4–6)	0 (0–4)	NA	7 (5–14)	NA

NA, not applicable; M, male, F, female; Temp, temperature (C) upon presentation to the ED; CRP, C-reactive protein (mg/l); WBC, white blood cell (10^9^/l); lymphocyte count expressed as 10^9^/l; X-ray, chest radiography results upon presentation to the ED; AB, adequate antibiotics within 12 hours after presentation; Duration of symptoms before ED presentation, ICU, Intensive Care Unit admission, LOS, length of stay.

### CRP Levels, WBC Counts, Lymphocyte Counts

On hospital admission (acute phase) of LD patients, median CRP level was 345 mg/l (range 256–455), median WBC count 12.0×10^9^/l (range 8.2–21.4) and median neutrophil count 10.5×10^9^/l (range 7.1–18.8). The median absolute lymphocyte count upon admission was 0.8×10^9^/l (range 0.4–1.6). In the subacute phase, median CRP level decreased significantly to 36 mg/l (range 10–59, p<0.05), whereas the median lymphocyte count increased significantly to 1.4×10^9^/l (range 0.8–3.4, p<0.05). The median WBC count of 10.3×10^9^/l (range 4.4–13.2) in the subacute phase did not differ from the acute phase of LD, whereas median neutrophil count was significantly decreased to 7.6×10^9^/l (range 2.7–8.6). In the age matched healthy controls, median CRP level, WBC count and neutrophil count were 2 mg/l (range 0–5), 7.1×10^9^/l (range 5.1–9.7) and 3.9×10^9^/l (range 3.1–6.3), respectively. All were significantly lower compared to the subacute phase of LD. The median lymphocyte count of 2.2×10^9^/l (range 1.4–3.0) in healthy controls did not differ from the subacute phase of LD (table 2).

**Table 2:** CRP levels, WBC counts, absolute neutrophil -, absolute lymphocyte - and lymphocyte subpopulation counts in the acute and subacute phase of Legionnares’ disease (n = 8) and in age and gender matched healthy controls (n = 8).

	Lymphocyte subpopulation markers	Acute phase of LD	Subacute phaseof LD	p-value*	Healthy Controls	p-value^#^
**CRP level**	NA	345 (256–455)	36 (10–59)	0.012	2 (0–5)	0.035
**WBC count**	NA	12.0 (8.2–21.4)	10.3 (4.4–13.2)	0.161	7.1 (5.1–9.7)	0.001
**Neutrophil count**	NA	10.5 (7.1–18.8)	7.6 (2.7–8.6)	0.018	3.9 (3.1–6.3)	0.015
**Lymphocyte count**	NA	0.8 (0.4–1.6)	1.4 (0.8–3.4)	0.012	2.2 (1.4–3.0)	0.115
**B-cells**	CD2^−^CD19^+^	148 (54–427)	177 (79–386)	0.401	165 (99–445)	0.753
**T-cells**	CD3^+^	495 (182–1024)	979 (507–2708)	0.012	1490 (870–2395)	0.462
**CD4^+^ T-cells**	CD3^+^CD4^+^	374 (146–629)	763 (400–1507)	0.012	947 (529–1500)	0.674
**Activated CD4^+^ T-cells**	CD3^+^CD4^+^CD38^+^HLA-DR^+^	10 (4–22)	70 (28–213)	0.012	28 (11–65)	0.016
**Naïve CD4^+^ T-cells**	CD3^+^CD4^+^CD45RA^+^	192 (56–445)	362 (197–819)	0.012	552 (286–844)	0.141
**Memory CD4^+^ T-cells**	CD3^+^CD4^+^CD45RA^−^	157 (53–232)	420 (203–756)	0.012	413 (203–721)	0.563
**CD8^+^ T-cells**	CD3^+^CD8^+^	119 (29–328)	224 (107–862)	0.012	434 (187–724)	0.189
**Activated CD8^+^ T-cells**	CD3^+^CD8^+^CD38^+^HLA-DR^+^	9 (4–43)	56 (15–154)	0.012	42 (4–97)	0.413
**Naïve CD8^+^ T-cells**	CD3^+^CD8^+^CD45RA^+^CD27^+^	61 (12–147)	79 (28–390)	0.012	227 (147–313)	0.197
**Memory CD8^+^ T-cells**	CD3^+^CD8^+^CD45RA^−^	25 (7–74)	116 (49–165)	0.012	128 (28–305)	0.600
**Effector CD8^+^ T-cells**	CD3^+^CD8^+^CD45RA^+^CD27^−^	15 (2–144)	26 (0–321)	0.058	50 (11–137)	0.439
**NK-cells**	CD3^−^CD16/56^+^	76 (26–171)	159 (91–262)	0.025	360 (221–623)	0.002

NA, not applicable; LD, Legionnaires’ disease; CRP, C-reactive protein (mg/l); WBC, white blood cell (10^9^/l); absolute neutrophil count expressed as 10^9^/l; NK, natural killer; absolute lymphocyte count expressed as 10^9^/l; lymphocyte subpopulation counts expressed as 10^6^/l; all data presented as median and range; *p-value Wilcoxon Signed Rank tests for differences in CRP levels, WBC counts, absolute neutrophil, absolute lymphocyte and lymphocyte subpopulation counts between the acute and subacute phase of LD; **^#^**p-value Mann Whitney U tests for differences between the subacute phase of LD and healthy controls.

### Flow Cytometric Analysis and Kinetics of Lymphocyte Subpopulations

Flow cytometric immunophenotyping of lymphocytes was performed on blood samples obtained in the acute and subacute phase of LD and from healthy controls (table 2). In the subacute phase of LD, significant increases compared to the acute phase were observed in CD3^+^ T-cell counts and CD3^−^CD16/56^+^ natural killer cell counts, but not in the number of CD2^−^CD19^+^ B-cells. Likewise, within the T-cells, significant increases were noted in both the CD3^+^CD4^+^ T-cell and CD3^+^CD8^+^ T-cell subpopulations. The CD4/CD8 ratio did not differ between the two time points (not shown).

**Figure 1:**
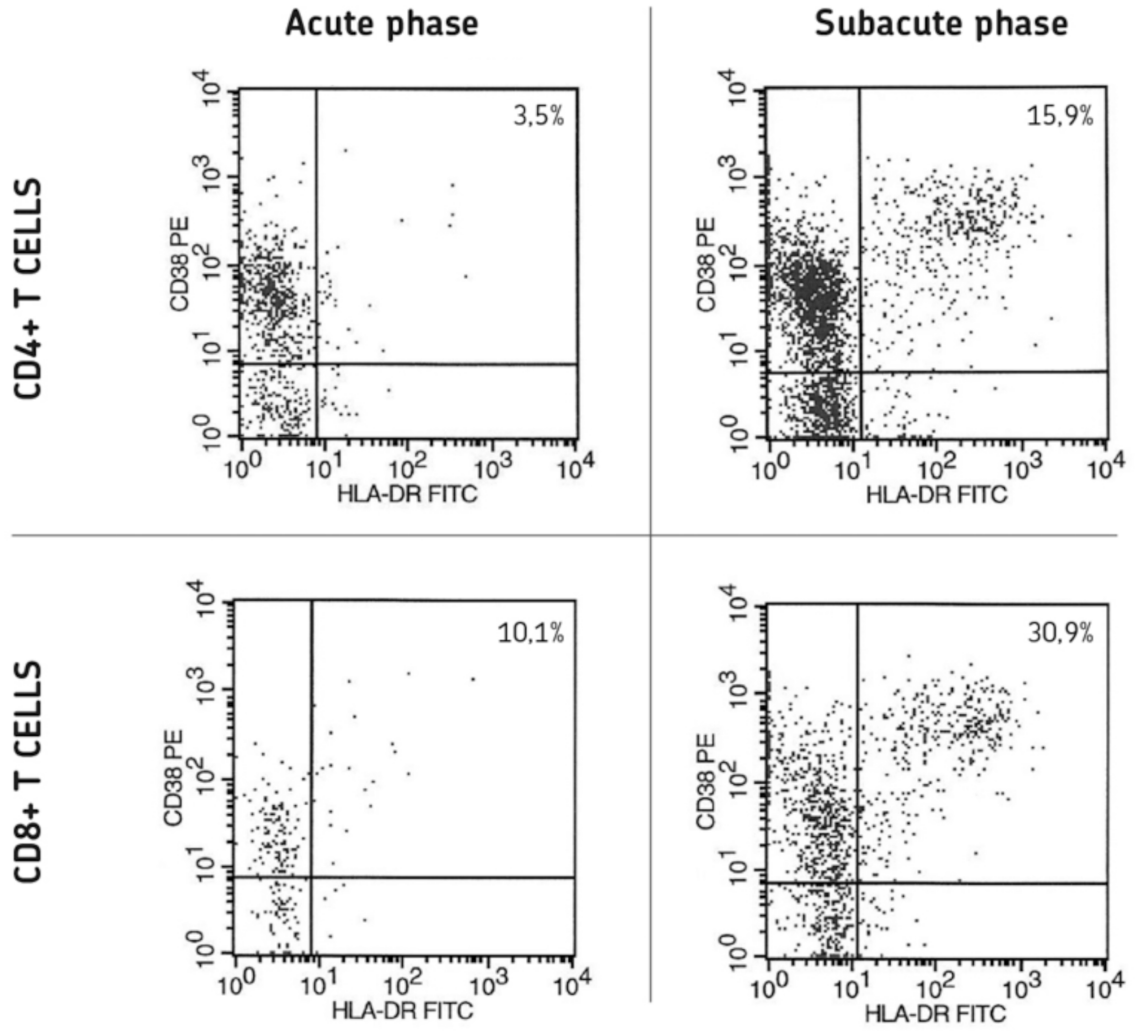
Flow cytometric analysis of activated CD4^+^ T-cells (top) and activated CD8^+^ T-cells (bottom) in the acute (left) and subacute phase (right) in one Legionnaires’ disease patient. Activated T-cells are defined as CD38+ and HLA-DR+ double positive cells and located in the upper right quadrants of each dot plot. The numbers represent the percentage of cells in the upper right quadrant.

Among the CD4^+^ T-cells, a significant increase in the absolute number of activated CD3^+^CD4^+^CD38^+^HLA-DR^+^ T-cells was observed in the subacute phase of LD compared to the acute phase. Likewise, a significant increase was noted for the number of activated CD3^+^CD8^+^CD38^+^HLA-DR^+^ T-cells. An example of the re-emergence of activated CD4^+^ T-cells and CD8^+^ T-cells in a single LD patient is shown in figure 1. In the CD4^+^ T-cell compartment, significant increases were furthermore observed for both the number of naïve CD3^+^CD4^+^CD45RA^+^ T-cells and memory CD3^+^CD4^+^CD45RA^−^ T-cells. Accordingly, among CD8^+^ T-cells, significant increases were noted in numbers of naïve CD3^+^CD8^+^CD45RA^+^CD27^+^ CD8^+^ T-cells and memory CD3^+^CD8^+^CD45RA^−^ T-cells, while effector CD3^+^CD8^+^CD45RA^+^ CD27^−^ T-cells did not show a significant difference between the acute and subacute phase of LD.

**Figure 2:**
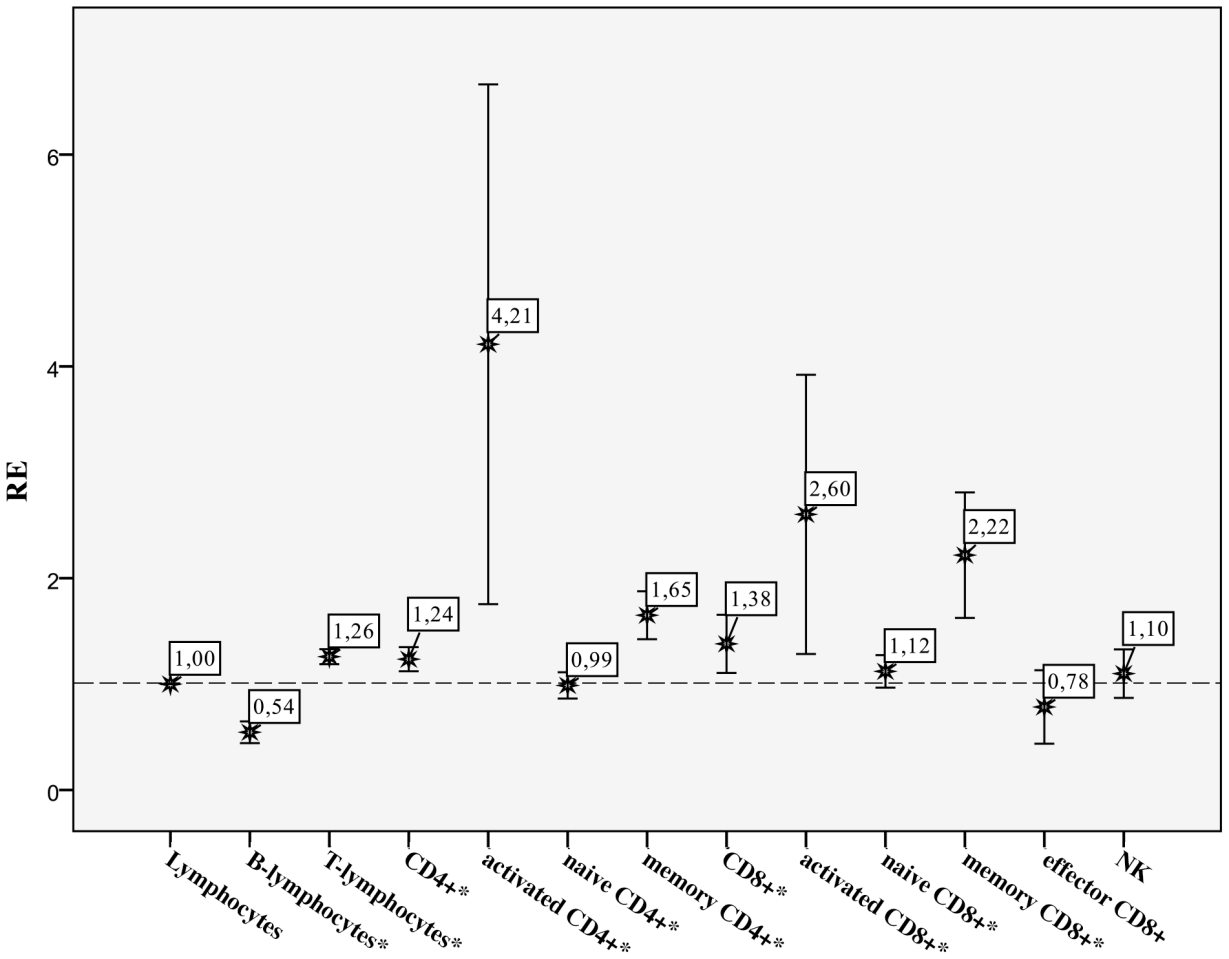
Relative expansion of lymphocyte subpopulations in the subacute phase compared to the acute phase of Legionnaires’ disease. RE, relative expansion: relative decrease or increase of the different absolute lymphocyte subpopulation counts in the acute versus the subacute phase compared to the relative increase of the absolute lymphocyte count in the same period; all data presented as mean and standard deviation; NK, natural killer cells; *Wilcoxon Signed Rank tests, significant difference p-value <0.05.

Finally, we compared the decrease or increase of the different lymphocyte subpopulations on the individual patient level in the acute versus the subacute phase to the relative change (increase) of the absolute lymphocyte count in the same period (acute versus subacute). In figure 2 this relative expansion of the different subpopulations is shown.

## Discussion

Lymphocytopenia has been observed in patients presenting with community-acquired LD and has recently been identified as a possible key diagnostic marker in this disease [22], [23]. Here, we confirm that the acute phase of LD is characterized by absolute lymphocytopenia. Furthermore, we show that lymphocyte counts recovered in the subacute phase as the clinical condition of the LD patients improved. The one patient with an adverse clinical course (intensive care unit admission, mechanical ventilation and longest length of stay) showed a delayed recovery of the lymphocyte count compared to the other LD patients (not shown).

Lymphocytopenia is observed in several infectious emergencies and can be used in the prediction of bacteremia in infectious emergency admissions [9], [10], [32]. The decrease in absolute lymphocyte count observed in critically ill patients is probably related to two factors: recruitment of lymphocytes from the bloodstream to peripheral tissues and accelerated apoptosis [16], [18], [33], [34], [35]. Although relatively unknown as a marker of disease severity or prognosis, lymphocytopenia has been described in several forms of CAP [11], [36]. Depression of absolute peripheral blood T-cell and T-cell subpopulation counts was observed in the majority of patients with respiratory infections. However, abnormalities in T-cell profiles did not predict outcome nor did it correlate with disease severity [11], [14]. Regarding CAP patients, it has been hypothesized that depression of absolute peripheral blood lymphocyte counts and lymphocyte T-cell counts reflects shifts of these cells towards the lungs, to be used in protective functions against the causative organism [11], [14], [15]. Absolute lymphocytopenia may result from T-cell migration to lung lesions and increased apoptosis of T-cells in *L. pneumophila* infections. Hypothetically, immune cells, including T-cells, may control inflammatory substances that are released from the initially infected site. The immune reaction to these substances, in case of affinity to lung cells, may be responsible for lung inflammation. During this process, non-specific T-cells against these substances may display increased apoptosis [37], [38].

Significant alterations in absolute peripheral blood CD4^+^ and CD8^+^ T-cell counts have been previously described in CAP patients and attributed to the severity of the underlying infection [11], [39]. To our knowledge the kinetics of lymphocyte subpopulations during LD have not been previously studied in detail. While the absolute B-cell count did not significantly change during the course of LD, we observed significant increases in absolute numbers of CD4^+^ and CD8^+^ T-cells. This is in agreement with the concept that cellular immunity is presumably essential for the resolution of *L. pneumophila* infection whereas humoral immunity plays a role only as a second line of defence by reducing the intrapulmonary bacterial multiplication [40], [41].

A/J mice inoculated intratracheally with *L. pneumophila* showed an increased number of circulating CD4^+^ and CD8^+^ T-cells in the second phase of infection when circulating cytokine levels and bacterial counts already declined [26], [42], [43]. T-cell depleted A/J mice showed impaired control of infection and increased lethality [26]. Furthermore, significant increases in the levels of the Th1 cytokines interferon-γ and interleukin-12 have been observed in the acute phase of LD emphasizing the importance of cell-mediated immunity in response to *L. pneumophila* infection [25].

Both activated CD4^+^ and activated CD8^+^ T-cell numbers were significantly decreased in the acute phase compared to the subacute phase of LD. Bronchoalveolar lavage findings of patients upon presentation with severe LD indicated a marked intrapulmonal increase of activated T-cells, defined as co-expression of HLA-DR and CD25, in combination with severe peripheral blood lymphocytopenia (mean absolute count 0.33×10^9^/l) [24]. Together, these observations illustrate that recruitment of (activated) lymphocytes from the bloodstream to the lung tissue could possibly explain the observed lymphocytopenia in the acute phase of LD. The recovery of absolute lymphocyte counts in the subacute phase of LD illustrates the reconstitution of the circulating lymphocyte pool. The relative expansion of lymphocyte subpopulations during LD was most prominent in the activated CD4^+^ T-cells, followed by the activated CD8^+^ T-cells. Further analysis also showed significant relative expansion of both memory CD4^+^ and memory CD8^+^ T-cell subpopulations but not of the naïve CD4^+^ T-cells subpopulation and both the naïve CD8^+^ and effector CD8^+^ T-cell subpopulations.

In the subacute phase of LD, most lymphocyte subpopulation counts showed no significant differences compared to matched healthy controls illustrating that patients in the subacute phase had significantly recovered. Correspondingly, CRP levels in the subacute phase were also clearly decreased compared to the acute phase. The numbers of activated CD4^+^ and activated CD8^+^ T-cells in the subacute phase of LD even exceeded values observed in matched healthy controls, reaching significance in case of activated CD4^+^ T-cells. Presumably, this observation reflects an overshoot of the lymphoproliferative response during recovery. The increased number of activated CD4^+^T-cells in the subacute phase compared to values obtained from healthy controls is probably due to the inflammatory response and the subsequent activation of these lymphocytes, which might be important for clearing of the infection or formation of long-term memory as it has been suggested that effector cells seed the memory pool [44].

This study has several limitations. First, we analysed a relatively small number of patients, which may affect statistical significance as some parameters have a wide range of values such as WBC count and differential. Second, we used a matched control group and although statistically there is a good association between distribution of lymphocyte subpopulations in the subacute phase of LD and the control patients, this may have introduced bias. Third, although the values of the peripheral blood lymphocyte subpopulations were comparable to reference values for peripheral blood lymphocyte phenotypes in healthy adults, differences in severity of illness and age between patients may have had impact on the degree of lymphocyte apoptosis and our results should be validated in a larger sample size [45]. Fourth, the subacute phase has been arbitrarily set on 7 days after the initial presentation of the patients. Three patients recovered earlier and therefore were discharged before the 7^th^ day. Blood samples on the day of discharge were used in these patients to assess the lymphocyte subpopulations. It is obvious that these patients showed better clinical improvement (also reflected by the decline of their CRP levels). Nevertheless, the different time-point of assessment of the lymphocyte subpopulations in the subacute phase could have introduced bias. Fifth, based upon our research, the exact role for the T-cell activation in LD remains to be delineated. Whether the observed changes in lymphocyte subpopulation numbers in our patients are attributed to redistribution, apoptosis or a combination of both remains to be determined. Neutrophils, monocytes and serum cytokine levels were not measured and both major pathways involved in the initiation of apoptosis, i.e., receptor initiated caspase-8-mediated pathway and mitochondrial-initiated caspase-9-mediated pathway were not analysed [46], .

### Conclusions

The acute phase of LD is characterized by absolute lymphocytopenia. This recovers in the subacute phase with an increase in absolute T-cell count and re-emergence of activated and memory CD4^+^ and CD8^+^ T-cells, following the possible recruitment of such cells to the lungs. These observations are in line with the suggested role for T-cell activation in the immune response to LD.
